# Obesity and the Microvasculature: A Systematic Review and Meta-Analysis

**DOI:** 10.1371/journal.pone.0052708

**Published:** 2013-02-06

**Authors:** Adrien Boillot, Sophia Zoungas, Paul Mitchell, Ronald Klein, Barbara Klein, Mohammad Kamran Ikram, Caroline Klaver, Jie Jin Wang, Bamini Gopinath, E. Shyong Tai, Aljoscha Steffen Neubauer, Serge Hercberg, Laima Brazionis, Seang-Mei Saw, Tien-Yin Wong, Sébastien Czernichow, META-EYE Study Group

**Affiliations:** 1 Department of Periodontology, University Paris 7, Paris, France; 2 Department of Nutrition, Ambroise Paré Hospital, Boulogne-Billancourt, France; 3 Department of Vascular Sciences and Medicine, The George Institute for Global Health, Victoria, Australia; 4 Department of Ophthalmology, Westmead Millennium Institute (Centre for Vision Research), Sydney, Australia; 5 Department of Ophthalmology and Visual Sciences, University of Wisconsin School of Medicine and Public Health, Madison, Wisconsin, United States of America; 6 Saw Swee Hock School of Public Health, National University of Singapore, Singapore, Singapore; 7 Department of Ophthalmology, Erasmus Medical Center, Rotterdam, The Netherlands; 8 Department of Ophthalmology, Singapore Eye Research Institute, Singapore, Singapore; 9 Department of Medicine, University of Melbourne, Victoria, Australia; 10 Department of Ophthalmology, Ludwig-Maximilians University, Muenchen, Germany; 11 Unité de Recherche en Épidémiologie Nutritionnelle, Institut National de la Recherche Agronomique/Institut National de la Recherche et de la Santé Médicale, Unité U557/Avicenne Hospital/Conservatoire des Arts et Métiers/Université Paris 13/Université Sorbone Paris Cité, Bobigny, France; 12 Department of Nutrition, Université Versailles St-Quentin, Versailles, France; 13 Institut National de la Recherche et de la Santé Médicale, Unité 1018, Centre for Research in Epidemiology and Population Health, Population-based Cohorts Research Platform, Villejuif, France; Zhongshan Ophthalmic Center, China

## Abstract

**Background:**

Overweight and obesity are thought to significantly influence a person's risk of cardiovascular disease, possibly via its effect on the microvasculature. Retinal vascular caliber is a surrogate marker of microvascular disease and a predictor of cardiovascular events. The aim of this systematic review and meta-analysis was to determine the association between body mass index (BMI) and retinal vascular caliber.

**Methods and Findings:**

Relevant studies were identified by searches of the MEDLINE and EMBASE databases from 1966 to August 2011. Standardized forms were used for data extraction. Among over 44,000 individuals, obese subjects had narrower arteriolar and wider venular calibers when compared with normal weight subjects, independent of conventional cardiovascular risk factors. In adults, a 1 kg/m^2^ increase in BMI was associated with a difference of 0.07 μm [95% CI: −0.08; −0.06] in arteriolar caliber and 0.22 μm [95% CI: 0.21; 0.23] in venular caliber. Similar results were found for children.

**Conclusions:**

Higher BMI is associated with narrower retinal arteriolar and wider venular calibers. Further prospective studies are needed to examine whether a causative relationship between BMI and retinal microcirculation exists.

## Introduction

Obesity is estimated by the World Health Organisation to affect 400 million people globally. Overweight/obesity is well recognized to increase a person's risk of cardiovascular disease (CVD) and this is possibly associated with the development of large vessel atherosclerosis in the carotid [1]–[3] and femoral [3] arterial beds. However, the impact of overweight/obesity on the microcirculation is less well described. Greater body-mass index (BMI) has been shown to result in a reduction in skin capillary density and lower tissue perfusion, one of the earliest detectable alterations in microvascular function in overweight individuals [4], [5]. Capillary recruitment is also reduced in obese individuals compared to lean control subjects [6].

The retinal microcirculation is accessible to non-invasive measurement and provides a window to the human microvasculature. Recent developments in retinal imaging techniques using computer-based analysis of retinal photographs allow quantification of retinal vascular caliber in an objective and reproducible way. Studies show that adverse changes in retinal vascular caliber (principally narrower retinal arteriolar caliber and wider venular caliber) are associated with cardiovascular risk factors, [7] the metabolic syndrome, [8] risk of diabetes, [9]–[10] hypertension, [11] coronary heart disease, [12] and stroke [13]–[14].

Several studies have reported an association between greater BMI and retinal vascular caliber. However, the consistency and strength of the associations have remained unclear. [15]–[16] Some studies suggest that greater BMI is associated with wider retinal venular caliber, while others report that greater BMI is associated with both narrower arteriolar and wider venular retinal calibers.

We conducted a systematic review and meta-analysis to determine the association between categories of BMI and retinal arteriolar and venular caliber, in adults and children. We examined whether these associations varied according to age and were independent of conventional cardiovascular risk factors.

## Methods

### Data sources and Searches

We performed a systematic review and meta-analysis of available literature according to the MOOSE guidelines. [17] To investigate the impact of BMI on retinal vascular parameters in humans (adults and children), relevant articles published in English between 1966 and August 2011 were identified from MEDLINE and EMBASE using a combined text and the following MeSH heading search strategies: (arteriolar narrowing OR retinal arteriolar caliber OR retinal venular caliber OR retinal vasculature OR retinal vascular caliber OR retina/arteriovenous ratio OR retinal microcirculation OR retinal vessels) AND (obesity OR BMI OR body mass index OR waist-to-hip ratio OR waist circumference) (Table S1). References listed in articles of interest were then scrutinised to identify other relevant studies and experts in the field were contacted to find additional relevant studies.

### Study selection

Studies were included if they had published quantitative estimates (including variability) of the association between body weight and central retinal arteriolar equivalent (CRAE) and/or central retinal venular equivalent (CRVE). In the case of duplicates, we included the most recent publication. Studies that were not published as full reports, such as conference abstracts or letters to editors, were excluded.

Titles of all articles retrieved from database searches were screened. The abstracts of relevant articles investigating the relationship between BMI and retinal vascular parameters were examined and all studies that could be included were retrieved. References from these studies and previous reviews were also scanned for any other relevant articles. The literature research was conducted by two authors (A.B. and S.C.) without disagreement over study eligibility.

### Data extraction and Quality Assessment

Due to the variety of reported outcomes and statistical methods (particularly variety of covariates included in multivariate analysis), authors of relevant studies for the meta-analysis were contacted by e-mail and asked to a complete a standardized data abstraction form regarding participants characteristics, and crude and adjusted retinal vascular parameters according to BMI categories.

### Outcome measurement

Retinal vascular diameters were assessed using 45-degree digital camera. Two photographic fields (optic disc and macula) were taken of each eye. Retinal vascular calibers were measured using computer-based programs. The right eye was used for assessment of retinal vascular calibre. If the photographs of the right eye were not gradable, the left eye assessment was used. For each image all arterioles and venules coursing through an area one-half to one disc diameter from the optic disc margin were measured and summarized as the central retinal arteriolar equivalent and the central retinal venular equivalent. Both represent the average diameter of arterioles and venules of the eye, respectively, by using a modification of the Parr-Hubbard formulas [18] as described by Knudtson et al [19].

### Data synthesis and analysis

Mean retinal vascular parameters were compared using mean differences (MD) across BMI categories (Underweight: <18.5 kg/m^2^, Normal weight: [18.5–24.9] kg/m^2^, Overweight: [25.0–29.9] kg/m^2^, Obese: ≥30 kg/m^2^) with [18.5–24.9] kg/m^2^ as the reference. Although BMI, age and gender dependent cut-offs exist for children, we chose the same categories for adults and children because we had no access to individual participant data. The inverse variance method with random effects was used. Moreover, crude and maximum adjusted regression coefficients were pooled to assess the continuous associations between retinal parameters and a 1kg/m^2^ increase in BMI. Significance was set at p<0.05 and 95% confidence intervals (95%CI) calculated. Analyses were conducted for both children and adults, with crude and maximum adjusted values of retinal outcomes (levels of adjustment are described for each included study in Table 1). The percentage of variability across studies attributable to heterogeneity rather than chance was estimated using the I^2^ statistic. [20] Publication bias was assessed using Egger's regression test. [21] Because of the number of studies included, we did not conduct subgroup analyses. All analyses were performed using R (R, version 2.12.1, the R Core Development team, 2010), STATA (Release 10; STATA Corporation, College Station, Texas, USA) and Review Manager (RevMan, version 5.0, The Cochrane Collaboration, 2008).

**Table 1:** Characteristics of studies included in the meta-analysis.

Study Ref	Cohort study	Country	Study size	Age (mean±sd)	Level of adjustment
Cheung N et al., 2007 (24)	Singapore Cohort Study of Risk Factors for Myopia (SCORM)	Singapore	1153	13.7±1.2	Age, gender, race, systolic blood pressure, diastolic blood pressure, eye measurements.
Gopinath B et al., 2011 (25)	Sydney Childhood Eye Study (SCES12yr)	Australia	2353	12.7±0.4	Age, gender, race, hypertension
Hughes A et al., 2009 (30)	Beaver Dam Eye Study cohort (BDES)	USA	4659	61.5±10.9	Age, gender, race, smoking, hypertension, type 2 diabetes mellitus, HDL cholesterol
Ikram M et al., 2004 (16)	Rotterdam Study	The Netherlands	5602	67.9±8.1	Age, gender, mean arterial blood pressure, non-fasting blood glucose, total cholesterol, smoking.
Jeganathan V et al., 2009 (28)	Singapore Prospective Study Program and Singapore Cardiovascular Cohort Study 2 (SPSP/SCCS2)	Singapore	3600	49.4±11.3	Age, gender, race, systolic blood pressure, smoking, cholesterol, HbA1c
Klein R et al., 2003 (23)	The Wisconsin Epidemiologic Study of Diabetic Retinopathy: XVIII (WESDR/T1DM)	USA	797	33.2±12.0	Age, gender, race, smoking, hypertension.
Klein R et al., 2006 (7)	The Wisconsin Epidemiologic Study of Diabetic Retinopathy: XX (WESDR/T2DM)	USA	1362	66.6±11.3	Age, gender, race, smoking, hypertension.
Liew G et al., 2008 (26)	Atherosclerosis Risk in Communities (ARIC)	USA	10778	59.7±5.6	Age, gender, race, diabetes medication, insulin use, glucose level, smoking, HDL, cholesterol, hypertension, medication for hypertension.
Sun C et al., 2008 (29)	Singapore Malay Eye Study (SIMES)	Singapore	3001	57.7±10.6	Age, gender, systolic blood pressure, HbA1c, smoking, cholesterol.
Taylor B et al., 2007 (9)	Sydney Childhood Eye Study (SCES6yr)	Australia	1773	6.7±0.4	Age, gender, race, hypertension.
Wang JJ et al., 2006 (27)	The Blue Mountains Eye Study (BMES)	Australia	3295	65.5±9.4	Age, gender, smoking, hypertension, type 2 diabetes mellitus.
Wong TY et al., 2006 (22)	The Multi-Ethnic Study of Atherosclerosis (MESA)	USA	5885	63.0±9.9	Age, gender, race, family history of type 2 diabetes mellitus, glucose level, insulin use, smoking, HDL, LDL, triglycerides, exercise, hypertension, medication for hypertension.

## Results

### Study characteristics

**Figure 1:**
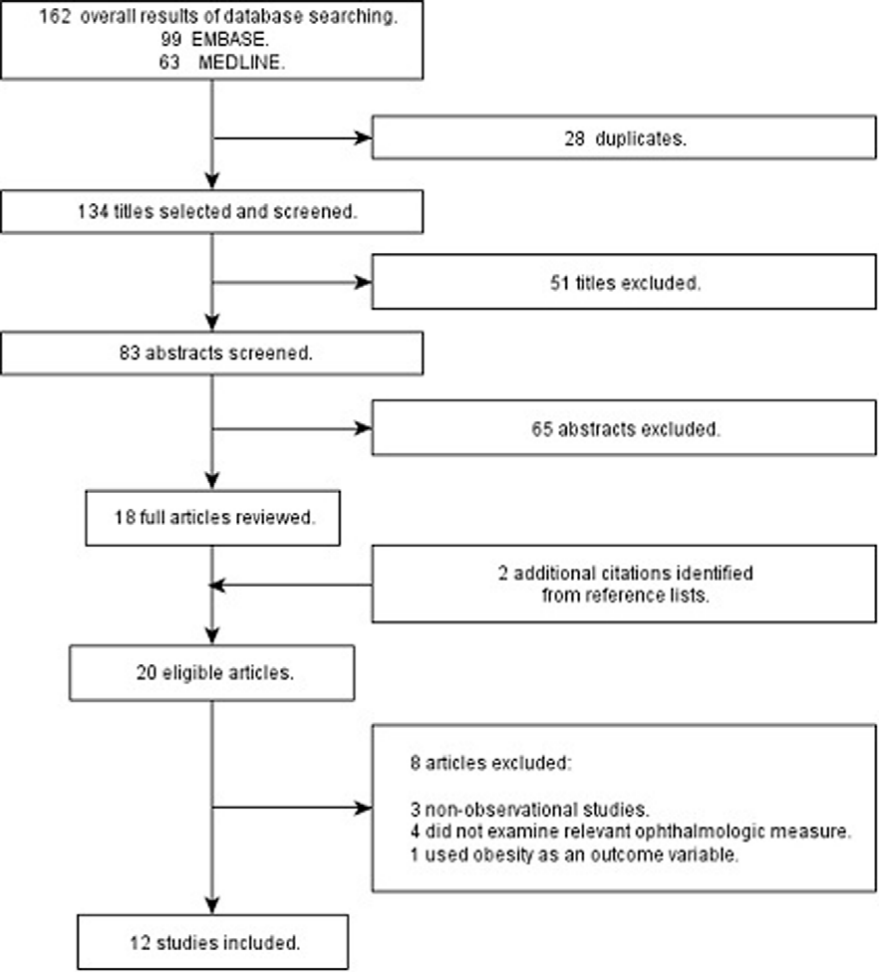
Flow-chart identifying eligible studies.

The search strategy identified a total of 162 titles including 28 duplicates and 51 titles that had no relevance to our primary research question. After reading the abstracts, the authors excluded 65 abstracts. Two additional studies were identified from reference lists. After review of 20 articles, 12 articles were selected and an email was sent to each contact author. [7], [9], [16], [22]–[30] All contacted authors contributed to the meta-analysis (Figure 1).

Nine studies were identified in adults [7], [16], [22], [23], [26]–[30] and three in children [9], [24], [25]. Sample sizes ranged from 797 [23] to 10,778 [26], for a total of 38,979 adults and 5,204 children. Study participants were from diverse countries including USA [7], [22]–[23], [26], [30], Australia [9], [25], [27], Singapore [24], [28]–[29] and The Netherlands [16] (Table 1). Mean BMI was 25.0 kg/m^2^ or higher in seven adult studies [7], [16], [22], [26]–[27], [29]–[30] In four studies [7], [27], [29]–[30] more than one-half of participants had hypertension and one study was conducted in people with type 2 diabetes [7] (Table S2).

### Mean differences in CRAE/CRVE across BMI categories

**Figure 2:**
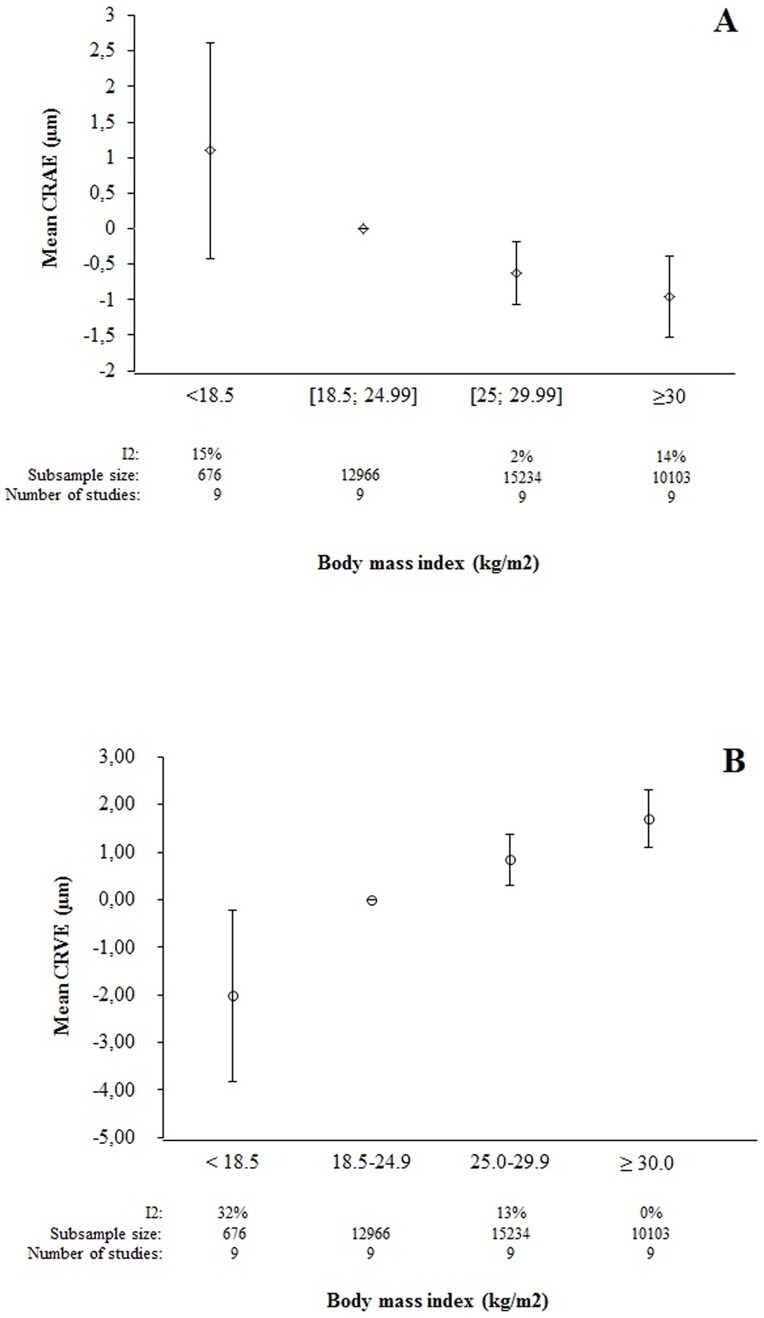
Adjusted adult mean retinal vascular values across BMI categories (maximum level of adjustment. CRAE (A); CRVE (B)).

**Figure 3:**
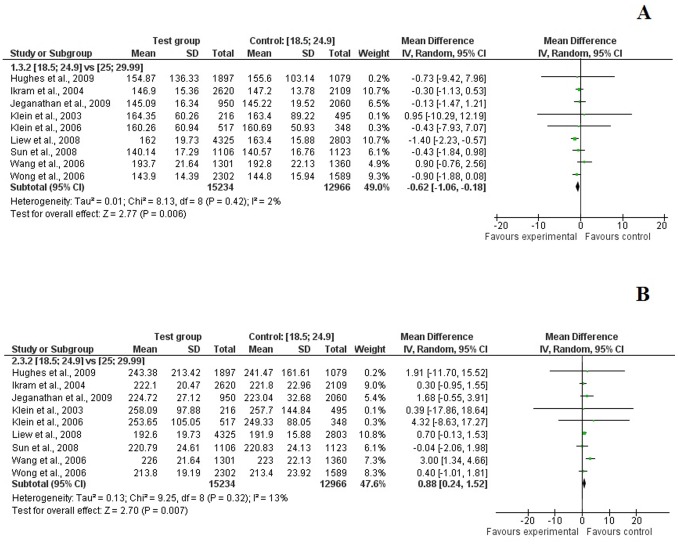
Association between overweight (BMI between 25.0 and 29.9 kg/m^2^) and CRAE (*A*), and CRVE (*B*) in adults (maximum level of adjustment).

**Figure 4:**
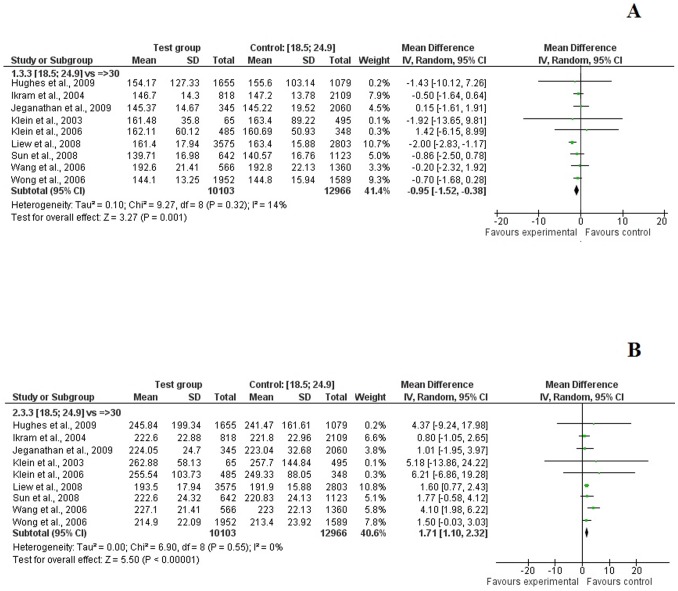
Association between obesity (BMI >30.0 kg/m^2^) and CRAE (*A*), and CRVE (*B*) in adults (maximum level of adjustment).

A higher BMI was associated with narrower arteriolar caliber in both adults and children (Figure 2, Table 2). When pooling maximum adjusted data, the mean difference was −0.62 μm [−1.06; −0.18] in overweight adults and −0.95 μm [−1.52; −0.38] in obese adults, when compared to their normal-weight counterparts (Figures 2, 3, 4). Similar results were found with children in crude (Table 2) and adjusted models (Table S3).

**Table 2:** Crude mean retinal vascular values across BMI categories.

	<18.5 vs [18.5; 24.99]		[18.5; 24.99] vs [25; 29.99]		[18.5; 24.99] vs ≥30	
	Mean difference (95% CI) (μm)	Heterogeneity (I^2^)	Mean difference (95% CI) (μm)	Heterogeneity (I^2^)	Mean difference (95% CI) (μm)	Heterogeneity (I^2^)
***Crude values. Adult studies^.(1)^***						
**CRAE**	3.25 [1.70, 4.80]^†^	22%	−1.04 [−1.62, −0.47]^†^	53%	−1.35 [−2.42, −0.28]^†^	82%
**CRVE**	−0.73 [−3.63, 2.16]	56%	1.54 [0.32, 2.76]^†^	81%	3.03 [1.04, 5.03]^†^	90%
***Crude values. Children studies.^(2)^***						
**CRAE**	1.89 [−1.13, 4.91]	87%	−1.44 [−2.90, 0.02]	56%	−1.59 [−4.18, 1.00]	63%
**CRVE**	0.31 [−3.26, 3.89]	82%	2.26 [0.90, 3.61]^†^	0%	6.54 [3.07, 10.02]^†^	62%

(1) *: p<0.05. †: p<0.01. <18.5 vs [18.5; 24.99]: n =  13642. [18.5; 24.99] vs [25; 29.99]: n =  28200. [18.5; 24.99] vs ≥30: n =  23069.

(2) *: p<0.05. †: p<0.01. <18.5 vs [18.5; 24.99]: n =  4004. [18.5; 24.99] vs [25; 29.99]: n =  4202. [18.5; 24.99] vs ≥30: n =  3647.

In contrast, the pooled summary estimates from studies of adults and children indicated that overweight and obese individuals had wider retinal venular caliber than their normal-weight counterparts (Figure 2, Table 2). After adjustment, overweight adults had a mean 0.88 μm [0.24; 1.52] increase in CRVE and 1.71 μm [1.10; 2.32] for obese adults (Figures 2, 3, 4). We found similar associations in children with a mean 2.20 [1.46; 2.95] increase in CRVE for overweight children and 6.73 μm [1.21; 12.25] for obese children (Table S3).

Testing revealed no evidence of heterogeneity among pooled studies of adults providing adjusted data for CRAE and CRVE in overweight (CRAE: I^2^ = 2%; CRVE: I^2^ = 13%) and obese (CRAE: I^2^ = 14%; CRVE: I^2^ = 0%). In contrast significant heterogeneity was observed among pooled studies of children providing adjusted data for CRAE in overweight (I^2^ = 75%) and for CRVE in obese (I^2^ = 91%).

No publication bias was observed (all p-values above 0.31 in adults and 0.09 in children).

### Continuous associations between CRAE/CRVE and BMI

In adults, a 1 kg/m^2^ increase in BMI was associated with a significant decrease in CRAE (−0.12 μm [−0.13; −0.11] in the crude model and −0.07 μm [−0.08; −0.06] in the adjusted model). Similar results were observed in children (−0.42 [−0.44; −0.40] in the crude model, and -0.20 [−0.23; −0.18] in the adjusted model).

A 1 kg/m^2^ increase in BMI was also associated with a significant increase in CRVE in adults (adjusted model 0.22 μm [0.21; 0.23]) and in children (adjusted model 0.28 μm [0.25; 0.30]).

## Discussion

This meta-analysis of over 44,000 individuals found that higher BMI levels were associated with narrower arteriolar and wider venular caliber, independent of conventional cardiovascular risk factors. This association was consistent across regions, ethnicity, age groups and study sample size. In studies of children significant heterogeneity in the pooled data was observed. This is likely to be explained by differences in the age range of the participants (two studies were conducted in children with mean age of 12–13 years [24], [25] and the third was conducted in children with mean age 6.7 years [9]), and the different levels of adjustment for confounding covariates.]. In spite of the heterogeneity in the pooled data for children, similar differences in retinal microvasculature were observed in adults and children with retinal arteriolar and wider venular calibres with increasing BMI, suggesting biological mechanisms involved in retinal microcirculation alterations are not age-dependent.

Our study suggests a significant influence of obesity on the human microcirculation. We offer several explanations for our findings. Nitric oxide (NO) is a key endothelium-derived relaxing factor causing vasodilatation and therefore an increase in volume perfusion in response to elevated metabolic demand. In obesity, decreased levels of NO have been observed and could explain impaired dilatation of the vasculature. [31] This endothelium-dependent dysfunction has been proposed to be responsible for the vasoconstriction observed in obese subjects. Moreover, increased levels of vasoconstrictor molecules (endothelin-1, angiotensin-II and other metabolites of arachidonic acid) have been associated with higher BMI. [32]–[33] Enhanced myogenic activation, mediated by antagonizism of calcium-activated potassium channels, has also been hypothesized to play a key role in alterations in blood flow in animal models of obesity [34]. In addition, a decrease in microvessel density, that may lead to tissue hypoxia and ischemia has been observed in obese subjects. Moreover, obesity-mediated insulin resistance may result in impaired capillary recruitment via insulin-mediated mechanisms [35].

Alterations in vascular stucture have previously been descibed in both animal and human models of obesity. These have included thickening of basement membranes, an increase in vascular diameter, stiffness of resistance arterioles and a decrease in lumen size. These changes lead to hypertension and increase the risk of ischemia, peripheral vascular disease and aneurysms [36].

Finally, excess release of inflammatory markers in obese subjects has been described and may contribute to vascular dysfunction. For example, TNFα is associated with impaired capillary recruitment and vasodilatation, and angiotensinogen is responsible for the activation of the renin-angiotensin system, resulting in higher production of angiotensin II, which is a powerful vasoconstrictor [37]. Vasoconstrictor and vasodilator mechanism alterations and anatomical changes result in impaired functional hyperemia in response to metabolic demand. Animal and human models have shown a reduced blood flow response to an increase in local metabolic activity in conditions of obesity [38].

An association between obesity and CVD such as coronary heart diseases, [39] stroke, [40] venous thromboembolism, [41] and even cardiovascular mortality [42] has long been described. More recently retinal caliber has also been shown to predict stroke [13] and coronary heart diseases [43]. Based on the results of our meta-analysis, we hypothesize that obesity induces microvascular alterations, reflected here in retinal vessel caliber. The clinical significance of these changes however, remain unclear. In children for example, the absolute differences in arteriolar and venular calibers between overweight and normal weight children were less than 1%, and whether these differences are clinically meaningful requires further study. If found to be clinically important retinal photography could be a useful non-invasive preventive tool to assess long-term microvascular changes as a marker of cardiovascular risk in overweight and obese persons. Review of longer-term inter-relationships between changes in obesity, retinal vessels and outcomes are now needed to determine whether changes are causally related to clinical outcomes.

The strengths of this meta-analysis are its large sample size and also the participation of all authors that were identified and contacted on the basis of the systematic search. Finally, because we were able to collect retinal vascular caliber data per BMI categories from each study, this reduced any heterogeneity in the categorisation of outcomes. This meta-analysis also has a number of limitations. First, an important limitation worth highlighting is the paucity of data on the prospective association between BMI and retinal vascular caliber, and also on the relationship between changes in BMI and in retinal vascular caliber over time. Consequently, a causal link cannot be extrapolated from these findings. Second, our study was limited to studies published in English, which results in a potential source of bias limiting the search to published reports. However, authors in this field were contacted to enhance the search field to grey litterature. Third, there is potential measurement bias due to different photographic procedures and software used in measuring retinal vascular caliber, resulting in possible overestimation or underestimation of the true association between BMI and retinal vascular calibers. Fourth, we standardized BMI categories according to the WHO classification for Caucasians whereas three studies were conducted in Asians [24], [28]–[29]. Also, both unmeasured and residual confounding, e.g. due to measurement error, may have influenced our findings. However, when regression coefficients were pooled to obtain a summary estimate corresponding to the change in retinal parameters for a 1 kg/m2 increase in BMI, results were still significant and had a similar level of magnitude. Fifth, because we had no access to individual participant data we used the same BMI categories for adults and children.

In conclusion, we observed in a large pooled population a significant association between overweight/obesity and narrower retinal arteriolar and wider venular caliber, while controlling for traditional cardiovascular risk factors (age, smoking, diabetes and hypertension). These findings suggest that obesity is not only linked to macrovascular pathology but also to microvascular changes. Further prospective studies are needed to explore a possible causative association between BMI and retinal microvasculature, which mirrors changes in the systemic microvasculature, including the microcirculation of the heart. If such a relationship is confirmed, examining retinal vascular caliber may provide information about small vessel alterations among individuals with obesity and other cardiovascular risk profiles. Small vessel disease is increasingly being recognized as a contributor to cardiovascular and cerebrovascular diseases, however, there is no widely available tool by which to assess small vessel condition. Retinal imaging and retinal vascular structural assessment may help to fill in this gap.

## Supporting Information

Table S1 **Sensitive search strategy.**
(DOC)

Table S2 **Description of studies included in the meta-analysis.**
(DOC)

Table S3 **Adjusted children mean retinal vascular values across BMI categories (maximum level of adjustment).** *: p<0.05. †: p<0.01. <18.5 vs [18.5; 24.99]: n =  4004. [18.5; 24.99] vs [25; 29.99]: n =  4202. [18.5; 24.99] vs ≥30: n =  3647.
(DOC)
