# Supplementary material for: Obesity and the Microvasculature: A Systematic Review and Meta-Analysis
Source: PLoS One. 2013 Feb 6;8(2):e52708. doi: 10.1371/journal.pone.0052708 (PMC3566162; doi:10.1371/journal.pone.0052708)
Supplement: Table S1 — Sensitive search strategy. (DOC) [file pone.0052708.s001.doc]

**Table S1**

| **Pubmed (63 results).** |
| --- |
| **#1** “retinal arteriolar narrowing”: 77 results.  **#2** “retinal arteriolar calibre”: 48 results.  **#3** “retinal arteriolar calibre”: 7 results.  **#4** “retinal venular caliber”: 36 results.  **#5** “retinal venular calibre”: 5 results.  **#6** “retinal vasculature”: 757 results.  **#7** “retinal vascular caliber”: 83 results.  **#8** “retinal vascular calibre” : 10 results.  **# 9** “retinal artery occlusion” [Mesh]: 1402 results.  **#10** “retinal vein occlusion” [Mesh]: 2417 results.  **#11** “retinal arteriovenous ratio”: 72 results.  **#12** “retinal venular dilatation”: 6 results.  **#13** “retinal microcirculation”: 195 results.  **#14** “retinal vessels” [Mesh]: 14187 results.  **#15**: #1 OR #2 OR #3 OR #4 OR #5 OR #6 OR #7 OR #8 OR #9 OR #10 OR #11 OR #12 OR #13 OR #14: 17414 results.  **#16** “Obesity” [Mesh]: 110665 results.  **#17** “Body Mass Index” [Mesh]: 58933 results.  **#18** “BMI”: 51690 results.  **#19** “Waist-Hip Ratio” [Mesh]: 2148 results.  **#20** “Waist Circumference” [Mesh]: 2026 results.  **#21** #16 OR # 17 OR # 18 OR #19 OR #20: 148342 results.  **#22** # 15 AND #21: 77 results.  **#23** #22 **Limits Activated:** Humans, English : 63 results. |
| **EMBASE (99 results).** |
| **#1** “retinal blood vessel’ [EMTREE]: 10443 results.  **#2** “retinal artery” [EMTREE]: 2060 results.  **#3** “retinal vein’ [EMTREE]: 1246 results.  **#4** “body mass index’ [EMTREE]: 114830 results.  **#5** “waist hip ratio’ [EMTREE]: 3843 results.  **#6** “waist circumference” [EMTREE]: 11444 results.  **#7** “obesity” [EMTREE]: 198741 results.  **#8** #4 OR #5 OR #6 OR #7:270891 results.  **#9** #1 OR #2 OR #3: 11251 results.  **#10** #8 AND #9: 127 results.  **#11** #10 AND [humans]/lim AND [english]lim: 99 results. |
