# Supplementary material for: Obesity and the Microvasculature: A Systematic Review and Meta-Analysis
Source: PLoS One. 2013 Feb 6;8(2):e52708. doi: 10.1371/journal.pone.0052708 (PMC3566162; doi:10.1371/journal.pone.0052708)
Supplement: Table S2 — Description of studies included in the meta-analysis. (DOC) [file pone.0052708.s002.doc]

**Table S2**

| **Study Ref** | **Cohort study** | **BMI**  **(kg/m^2^,mean + sd)** | **Gender (Male, %)** | **Current smokers**  **(%)** | **Race** | |
| --- | --- | --- | --- | --- | --- | --- |
|  |  |  |  |  | **Whites (%)** | **Asians (%)** |
| Cheung N et al., 2007 ^25^ | Singapore Cohort Study of Risk Factors for Myopia (SCORM) | 19.60+4.00 | 48.74 | -- | -- | 99.31 |
| Gopinath B et al., 2011 ^26^ | Sydney Childhood Eye Study  (SCES12yr) | 20.40+4.20 | 50.50 | -- | 59.80 | 15.00 |
| Hughes A et al., 2009 ^31^ | Beaver Dam Eye Study cohort (BDES) | 28.71± 5.25 | 44.07 | 20.26 | 99.40 | 0.26 |
| Ikram M et al., 2004 ^17^ | Rotterdam Study | 26.30+3.68 | 41.00 | 24.00 | 100.00 |  |
| Jeganathan V et al., 2009 ^29^ | Singapore Prospective Study Program and Singapore Cardiovascular Cohort Study 2 (SPSP/SCCS2) | 24.13+4.37 | 48.10 | 12.00 | -- | 100.00 |
| Klein R et al., 2003^24^ | The Wisconsin Epidemiologic Study of Diabetic Retinopathy : XVIII (WESDR/T1DM) | 24.36± 4.12 | 50.19 | 29.11 | 98.86 | -- |
| Klein R et al., 2006 ^8^ | The Wisconsin Epidemiologic Study of Diabetic Retinopathy : XX (WESDR/T2DM) | 28.70 ± 5.63 | 46.48 | 14.24 | 98.46 | -- |
| Liew G et al., 2008 ^27^ | Atherosclerosis Risk in Communities (ARIC) | 28.50+5.50 | 44.50 | 20.70 | 79.40 | -- |
| Sun C et al., 2008 ^30^ | Singapore Malay Eye Study (SIMES) | 26.44+5.12 | 48.20 | 20.80 | -- | 100.00 |
| Taylor B et al., 2007 ^10^ | Sydney Childhood Eye Study (SCES6yr) | 16.20+2.10 | 50.70 | -- | 63.70 | 17.10 |
| Wang JJ et al., 2006 ^28^ | The Blue Mountains Eye Study (BMES) | 26.20 ± 4.52 | 43.60 | 15.10 | 99.00 | -- |
| Wong TY et al., 2006 ^23^ | The Multi-Ethnic Study of Atherosclerosis (MESA) | 28.4+5.50 | 47.80 | 11.70 | 39.80 | 11.70 |

**Table S2 (*Cont*):**

| **Study Ref** | **Systolic blood pressure**  **(mm Hg, mean + sd)** | **Diastolic blood pressure**  **(mm Hg, mean + sd)** | **Hypertension (%)** | **Type 2 diabetes mellitus**  **(%)** | **Total cholesterol**  **(mg.dl^-1^, mean + sd)** | **LDL cholesterol**  **(mg.dl^-1^, mean + sd)** |
| --- | --- | --- | --- | --- | --- | --- |
| Cheung N et al., 2007 ^25^ | 110.20**+**13.76 | 63.99**+**8.84 | -- | -- | -- | -- |
| Gopinath B et al., 2011 ^26^ | 112.70**+**10.90 | 65.10**+**8.60 | 26.80 | -- | -- | -- |
| Hughes A et al., 2009 ^29^ | 131.90 ± 20.28 | 77.48 ±10.87 | 50.09 | 10.28 | 234.23±43.84 | 52.16±17.68 |
| Ikram M et al., 2004 ^18^ | 138.50**+**22.10 | 73.70**+**11.40 | 33.00 | 10.00 | 256.77**+**46.40 | -- |
| Jeganathan V et al., 2009 ^31^ | 131.64**+**20.24 | 77.85**+**10.71 | 39.90 | 10.30 | 202.24**+**36.35 | 124.13**+**32.87 |
| Klein R et al., 2003 ^27^ | 127.57±21.33 | 80.00±11.46 | 25.79 | 0.00 | -- | -- |
| Klein R et al., 2006 ^33^ | 147.46 ±24.00 | 79.18 ±12.00 | 58.27 | 100.00 | -- | -- |
| Liew G et al., 2008 ^28^ | 123.9**+**18.70 | 71.80**+**10.40 | 39.60 | 9.00 | 207.60**+**37.70 | 52.10**+**18.20 |
| Sun C et al., 2008 ^32^ | 145.91**+**23.14 | 79.62**+**11.10 | 67.10 | 23.50 | 216.94**+**44.47 | 136.51**+**38.67 |
| Taylor B et al., 2007 ^10^ | 99.70**+**10.60 | 60.10**+**10.40 | 17.30 | -- | -- | -- |
| Wang JJ et al., 2006 ^30^ | 145.80±21.4 | 83.40± 10.10 | 70.80 | 7.60 | 232.80±41.7 | 146.40±38.00 |
| Wong TY et al., 2006 ^8^ | 123.9 (20.7) | 70.50**+**10.10 | 45.10 | 14.40 | 191.5**+**35.80 | 113.80**+**32.10 |

| **Study Ref** | **Systolic blood pressure**  **(mm Hg, mean + sd)** | **Diastolic blood pressure**  **(mm Hg, mean + sd)** | **Hypertension (%)** | **Type 2 diabetes mellitus**  **(%)** | **Total cholesterol**  **(mg.dl^-1^, mean + sd)** | **LDL cholesterol**  **(mg.dl^-1^, mean + sd)** |
| --- | --- | --- | --- | --- | --- | --- |
| Cheung N et al., 2007 ^25^ | 110.20**+**13.76 | 63.99**+**8.84 | -- | -- | -- | -- |
| Gopinath B et al., 2011 ^26^ | 112.70**+**10.90 | 65.10**+**8.60 | 26.80 | -- | -- | -- |
| Hughes A et al., 2009 ^29^ | 131.90 ± 20.28 | 77.48 ±10.87 | 50.09 | 10.28 | 234.23±43.84 | 52.16±17.68 |
| Ikram M et al., 2004 ^18^ | 138.50**+**22.10 | 73.70**+**11.40 | 33.00 | 10.00 | 256.77**+**46.40 | -- |
| Jeganathan V et al., 2009 ^31^ | 131.64**+**20.24 | 77.85**+**10.71 | 39.90 | 10.30 | 202.24**+**36.35 | 124.13**+**32.87 |
| Klein R et al., 2003 ^27^ | 127.57±21.33 | 80.00±11.46 | 25.79 | 0.00 | -- | -- |
| Klein R et al., 2006 ^33^ | 147.46 ±24.00 | 79.18 ±12.00 | 58.27 | 100.00 | -- | -- |
| Liew G et al., 2008 ^28^ | 123.9**+**18.70 | 71.80**+**10.40 | 39.60 | 9.00 | 207.60**+**37.70 | 52.10**+**18.20 |
| Sun C et al., 2008 ^32^ | 145.91**+**23.14 | 79.62**+**11.10 | 67.10 | 23.50 | 216.94**+**44.47 | 136.51**+**38.67 |
| Taylor B et al., 2007 ^10^ | 99.70**+**10.60 | 60.10**+**10.40 | 17.30 | -- | -- | -- |
| Wang JJ et al., 2006 ^30^ | 145.80±21.4 | 83.40± 10.10 | 70.80 | 7.60 | 232.80±41.7 | 146.40±38.00 |
| Wong TY et al., 2006 ^8^ | 123.9 (20.7) | 70.50**+**10.10 | 45.10 | 14.40 | 191.5**+**35.80 | 113.80**+**32.10 |
