# Supplementary material for: Obesity and the Microvasculature: A Systematic Review and Meta-Analysis
Source: PLoS One. 2013 Feb 6;8(2):e52708. doi: 10.1371/journal.pone.0052708 (PMC3566162; doi:10.1371/journal.pone.0052708)
Supplement: Table S3 — Adjusted children mean retinal vascular values across BMI categories (maximum level of adjustment). *: p<0.05. †: p<0.01. <18.5 vs [18.5; 24.99]: n = 4004. [18.5; 24.99] vs [25; 29.99]: n = 4202. [18.5; 24.99] vs ≥30: n = 3647. (DOC) [file pone.0052708.s003.doc]

**Table S3**

|  | **<18.5 vs [18.5; 24.99]** | | **[18.5; 24.99] vs [25; 29.99]** | | **[18.5; 24.99] vs ≥30** | |
| --- | --- | --- | --- | --- | --- | --- |
|  | **Mean difference (95% CI)** | **Heterogeneity (I^2^)** | **Mean difference (95% CI)** | **Heterogeneity (I^2^)** | **Mean difference (95% CI)** | **Heterogeneity (I^2^)** |
|  | ***Adjusted values. Children studies.*** | | | | | |
| **CRAE** | 1.05 [-0.98, 3.07] | 75% | -1.44 [-3.47, 0.58] | 75% | -1.38 [-2.36, -0.40]^§^ | 10% |
| **CRVE** | -1.14 [-3.15, 0.87] | 55% | 2.20 [1.46, 2.95]^§^ | 0% | 6.73 [1.21, 12.25]^*^ | 91% |

^*^: p<0.05. ^§^: p<0.01 . <18.5 vs [18.5; 24.99]: n= 4004. [18.5; 24.99] vs [25; 29.99]: n= 4202. [18.5; 24.99] vs ≥30: n= 3647.
